# Assessment of fishery resources using environmental DNA: Small yellow croaker (*Larimichthys polyactis*) in East China Sea

**DOI:** 10.1371/journal.pone.0244495

**Published:** 2020-12-29

**Authors:** Xiaoyan Wang, Guoqing Lu, Linlin Zhao, Qiao Yang, Tianxiang Gao

**Affiliations:** 1 National Engineering Research Center of Marine Facilities Aquaculture, Zhejiang Ocean University, Zhoushan, PR China; 2 Department of Biology, University of Nebraska at Omaha, Omaha, Nebraska, United States of America; 3 First Institute of Oceanography Ministry of Natural Resources, Qingdao, PR China; 4 ABI Group of GPM Project, Zhejiang Ocean University, Zhoushan, PR China; Tanzania Fisheries Research Institute, UNITED REPUBLIC OF TANZANIA

## Abstract

Species distribution monitoring and biomass assessment are crucial for fishery management and resource conservation. However, traditional methods such as motor trawling are costly and less effective than the novel environmental DNA (eDNA) approach. This study employs eDNA approach to investigate horizontal and vertical distributions of small yellow croaker (*Larimichthys polyactis*), an economically important species, in the East China Sea. The analysis of 171 eDNA samples collected from 44 stations using the species-specific primers and Taqman probe suggests a presence of small yellow croaker at 28 sampling layers in 44 stations. Significant differences in croaker eDNA concentrations were revealed among sampling stations and layers, consistent with previous findings through motor-trawl capture offshore and nearshore ichthyoplakton surveys, indicating small yellow croaker exhibits strong regional distribution and layer preference. In addition, we found a high eDNA concentration of small yellow croaker in the surface waters beyond the motor-trawl prohibition line, which confirms spawning grounds have been expanded from nearshore to offshore areas. Such expansion of spawning grounds could be a response by small yellow croaker to stressors such as overfishing, climate change, and nearshore environment contamination. To identify environmental variables potentially associated with small yellow croaker presence and absence, we conducted a correlation analysis between eDNA concentration and environmental variables, and the results provide a guideline for further investigation of fishery resources in the future. In conclusion, this study demonstrates the power of the eDNA approach in monitoring small yellow croaker at extensive geographic scales. The developed protocols and the findings are expected to assist in long-term monitoring and protection programs and benefit sustainable fishery in small yellow croaker.

## Introduction

Effective fishery management and biodiversity conservation require accurate information on spatial distribution and population status [1, 2]. Small yellow croaker *(Larimichthys polyactis*), an important demersal, warm-temperature fish species, is widely distributed in the coastal areas of the Bohai Sea, Yellow Sea, and East China Sea [3–5]. It is a commercially important fish supporting the demersal fisheries of Korea, Japan and China [3, 4]. Small yellow croaker regularly migrates among spawning, feeding, and overwintering grounds. The spawning time of small yellow croaker runs from March to June, with a peak of pelagic eggs in the coastal waters of the East China Sea and the Yellow Sea in April to May [3]. Three geographical stocks (i.e., northern, Lvsi, and East China Sea) of the croaker were identified based on spawning migration patterns and morphological characters [3]. The small yellow croaker had been under high fishing pressure and over-exploitation for decades, including phases of prosperity (1950–1960), decline (1961–1976), nearly collapsing (1977–1989) [6]. Due to the implementation of various management and conservation measures, small yellow croaker natural resources have been gradually recovering since the 1990s [6].

The small yellow croaker and large yellow croaker (*Larimichthys crocea*) belong to the genus *Larimichthys* and are morphologically similar. The two fish species share spawning and feeding grounds in the East China Sea and the southern Yellow Sea. They were once considered two of the four major fish species in traditional marine fisheries in China. Due to overexploitation and habitat degradation, the fishery resources of large yellow croaker were nearly depleted. The total catch for *L*. *polyactis* in the 1950s was 5 to 10×10^4^ tons annually, which is considered overfishing [7]. After a decline and collapse period during 1961–1989, annual capture on average reached 400,000 tons during 2006–2016 (http://www.fao.org/fishery/species/2362/en). Although the small yellow croaker fishery has been recovered since the 1990s, the population and biological characters were found to have changed, including the decrease of body size, early maturation, and the dispersal of spawning grounds [4, 6, 8–10]. For example, the spawning grounds have expanded from nearshore in the past to offshore at present [3]. In addition, spatial and temporal distributional changes in fish habits and migration patterns were observed in previous studies [11, 12]. To avoid possible population depletion like the large yellow croaker, it is essential to take effective measures to protect small yellow croaker resources [13].

Traditional fishery assessment methods such as trawling have been performed routinely for fisheries monitoring [14]. However, these methods are generally time-consuming, costly, and environmentally unfriendly and require taxonomic expertise for species classification [15]. In addition, traditional methods are not applicable in particular times (such as the summer fishing moratorium period) and particular areas (such as the forbidden fishing zone or conservation area). Importantly, traditional fishery assessment methods may, in some cases, fail to provide necessary data for fishery management [1]. New noninvasive, accurate, effective, and environmentally friendly methods are therefore required [16, 17]. Environmental DNA (eDNA) is the DNA extracted directly from environmental samples (e.g., sediment, feces, water, soil, or air) without the need for intervening of a particular organism [18, 19]. Growing evidence has shown that the quantity of eDNA was related not only to species presence or absence but also to species abundance [14, 20–23]. Positive correlations between eDNA and biomass have been verified in previous studies, including ponds and lakes, rivers and streams, and oceans [20–23]. Environmental DNA has also been applied to investigate fish reproductive migration [22, 24, 25]. For example, the Shishamo smelt *Spirinchus lanceolatus* eDNA was consistently detected throughout the spawning season in a spawning migration core river, and the temporal distribution of eDNA concentration was consistent with that of the number of migrating *S*. *lanceolatus* estimated by catch survey data [26]. Besides eDNA fishery assessment in freshwater, the mystery spawning region of the Japanese eel *Anguilla japonica* was eventually discovered and validated by eDNA surveys [22]. The eDNA method has great potential in fisheries management, especially suitable for large-scale and high-frequency sampling without habitat disturbances [14].

In this study, we developed small yellow croaker-specific primers and probe for the detection of small yellow croaker. The eDNA approach was applied to investigate horizontal and vertical distributions of small yellow croaker and identify key environmental factors likely associated with its distribution. This study is expected to contribute to the assessment of stocking strategies in fisheries enhancement and provide a practical protocol for resource surveys on other aquatic species.

## Materials and methods

### Small yellow croaker-specific primers and probe development

The *L*. *polyactis* specific primers and probe set were designed and confirmed *in silico*, *in vitro*, and *in situ*. Specifically, primers and the probe were designed by comparing complete mitochondrial DNA sequences of Sciaenidae family fish species using primer designing tool (https://www.ncbi.nlm.nih.gov/tools/primer-blast/), SeqMan, and Primer Express 3.0.1. The designed primers and probe were confirmed by a CFX96 Touch Real-Time PCR Detection System (Bio-Rad, Hercules, CA) using the genome DNA of Sciaenidae family fish species and artificial mixed DNA as templates. Consequently, a pair of primers (forward: 5′-AGAGGCCCAAGTCGATAGTCAAC-3′ and reverse: 5′-CTTCGGGTGCGTATAACAGCTT-3′) and *L*. *polyactis* -specific TaqMan^™^ minor-groove-binding probe (5′-HEX- TTAGATAGAACCCAAAACTAAAG-3′MGB) were developed (Sangon Biotech, Shanghai, China). Plasmid standard was prepared by insert amplified *L*. *polyactis* products into a pESI-T vector. Series dilutions (10^1^ to 10^7^ copies/ μL) of plasmid standard were used as RT-PCR standards, and the detection limits were 2.67 for *L*. *polyactis*. Negative filtration controls, negative DNA extraction controls, and no template control (sterile water in place of DNA) were included on each RT-PCR plate. All samples were assayed in triplicate.

### Field sampling

**Fig 1:**
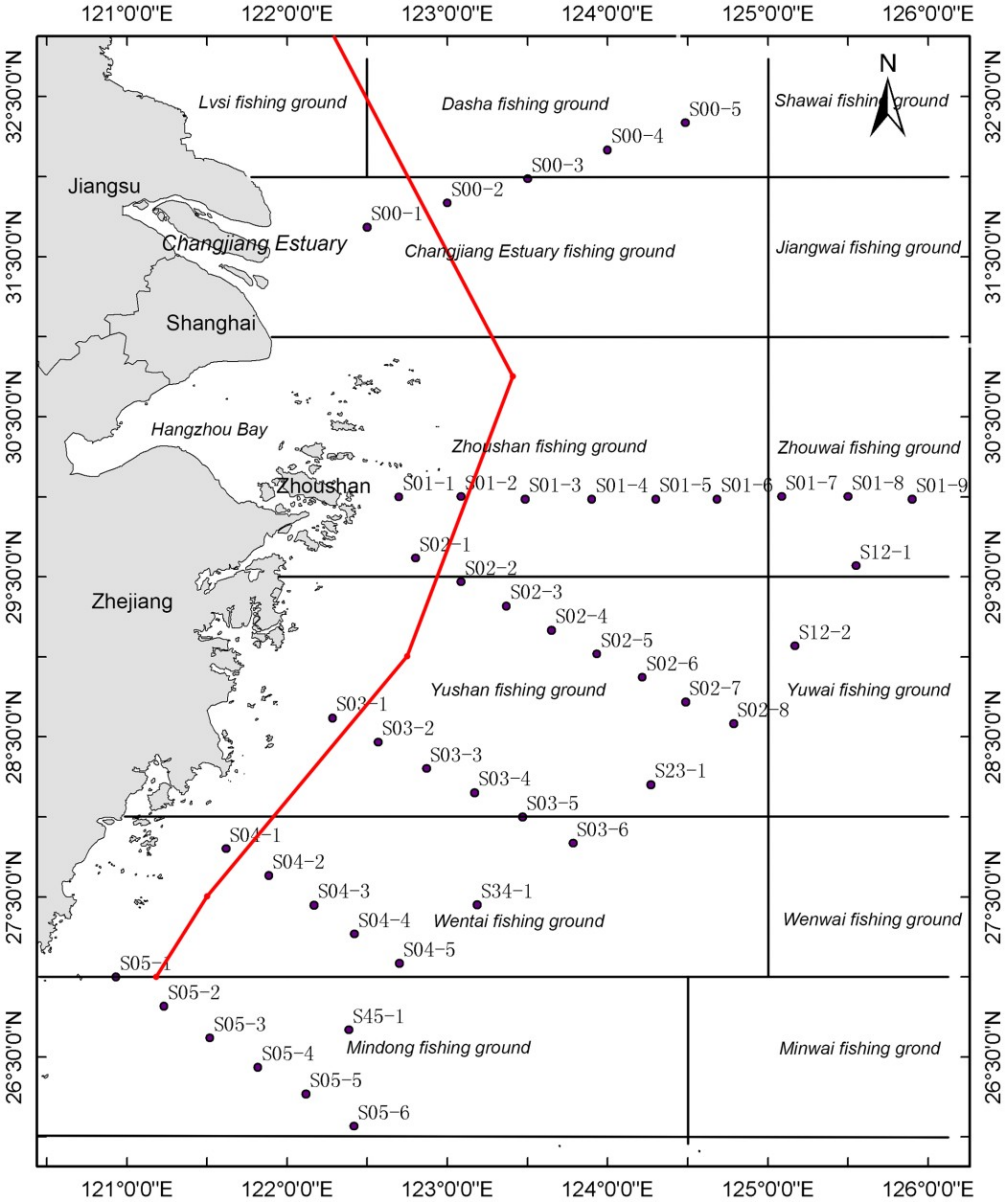
Map of sampling stations (black dots) of the East China Sea with adjacent provinces (Jiangsu and Zhejiang), a municipality (Shanghai), and fishing grounds (e.g., Zhoushan fishing ground) denoted. The red line indicates the nearshore areas where fishing is banned with motor trawlers.

A total of 171 samples from 44 sampling stations were collected from May 14 to 24, 2019, in the East China Sea (Fig 1). Seawater samples were collected using 24×20L Niskin bottles with SBE 911 Plus CTD system (Sea Bird Scientific, USA), and seawater from each depth was kept in a disposable sterile plastic bag before filtering. One liter of seawater from each sample was filtered onto a glass fiber (47 mm diameter with a pore size of 0.45 um). Filter funnels were washed with tap water, soaked in a sodium hypochlorite solution for 10 min, washed with tap water, and finally with distilled water before each sample was filtered. New gloves were changed for each sample. Distilled water and tap water were used as negative controls. All filters preserved by freezing at -196 °C in liquid nitrogen were taken to the laboratory and stored at -80 °C until further eDNA extraction.

### Environmental DNA assay

The frozen filters were thawed on ice. Environmental DNA was extracted from the filter using a DNeasy Blood & Tissue kit (Qiagen, Hilden, Germany) following the manufacturer’s protocol and the methods of Miya et. al. [27]. OneStep PCR Inhibitor Removal Kit (Zymo Research, USA) was used to remove the inhibitors [28]. Real-time PCR was conducted through two-step cycling protocols. TaqMan Fast qPCR Master Mix (Sangon, Shanghai, China) was used following the manufacturer’s protocol. A negative control (pure water as a template) was also analyzed with the same protocol to monitor contamination during filtration, eDNA extraction, and RT-PCR. All samples were assayed in triplicate.

### Environmental variables

Hydrographic data, such as total depth, water sampling depth, temperature, salinity, oxygen, and turbidity, were recorded by the CTD system, whereas other environmental variables, such as chlorophyll and nutrients (PO_4_-P, NH_4_-N, SiO_3_-Si, NO_3_^-^, and NO_2_^-^) were determined by the First Institute of Oceanography Ministry of Natural Resources.

### Statistical analyses

The continental shelf distribution of the East China Sea can be roughly divided into an inner shelf and an outer shelf based on the 50-60m isobaths, and the highest sampling depth is about 100 m, so 50m was selected as a depth boundary in current study. Three layers based on the actual water depth (i.e., Layer1: surface water; layer2: 10–50 m; and layer3: 50 m to 104 m) were used to examine whether there is a vertical effect of sampling water depths on eDNA. Statistical differences of eDNA concentration among three layers and stations were assessed using the Kruskal-Wallis test.

The correlation between environmental variables and logarithm transformed eDNA concentrations (i.e., ln([eDNA]+1)) was determined using Pearson’s method in “PerformanceAnalytics” package.

Multiple linear regression (MLR) was used to determine the best fitting models for small yellow croaker biomass based on environmental variables. Preliminary analyses were conducted to ensure no violation of the assumptions of normality, linearity, and homoscedasticity. The eDNA concentrations were *Ln* transformed to conform to a normal distribution prior to analysis. Within the MLR model, the 12 environmental variables (i.e., total depth, water sampling depth, temperature, salinity, oxygen, turbidity, PO_4_-P, NH_4_-N, SiO_3_-Si, NO_3_^-^, and NO_2_^-^) were included as independent variables, ln([eDNA]+1) was included as the dependent variables. The MLR was performed using all subsets regression methods using the “Leaps” package [29]. The MLR model was validated using Adjusted Akaike’s Information Criterion (AIC) and “gvlma” package. Co-linearity between predictive variables was assessed with the variance inflation factor (VIF), and co-variables with VIF greater than 5, removed.

We also conducted a principal component analysis ordination based on the environmental variables measured at the 171 seawater samples from 44 sampling sites to visualize whether eDNA presence/absence samples are separated, and identify variables likely associated with eDNA concentrations. The eDNA concentrations were transformed into presence or absence data prior to the PCA analysis. The “psych” package was applied for PCA analysis.

All statistical analyses were performed using R software version 3.5.3 and SPSS 21.0. The sampling stations and spatial distribution of *L*. *polyactis* were visualized with ArcGIS 10.1 and Ocean Data view 4 software.

## Results and discussion

### The specificity of the designed primer/probe set confirmation

We designed small yellow croaker specific primers and probe. We cross-validated the primers and the probe using a closely related fish, *L*.*croea*, which belongs to the same genus of *Larimichthys* with small yellow croaker, though i*n silico* sequence analysis and *in vitro* real-time PCR. The primers can amplify both species’ DNA, however the Taqman probe was found specific to the small yellow croaker. Six-nucleotide differences between the target sequences of the probe in the two fish species warrant the species-specific detection of small yellow croaker. The *in vitro* specificity test on large yellow croaker’s DNA revealed no amplification signal. The plasmid standards of small yellow croaker showed amplification signals with Ct values between 14.0 and 37.6, with no amplification signals observed in PCRs using non-target species DNA as template or negative controls.

### Horizontal distribution of *L*. *polyactis*

**Fig 2:**
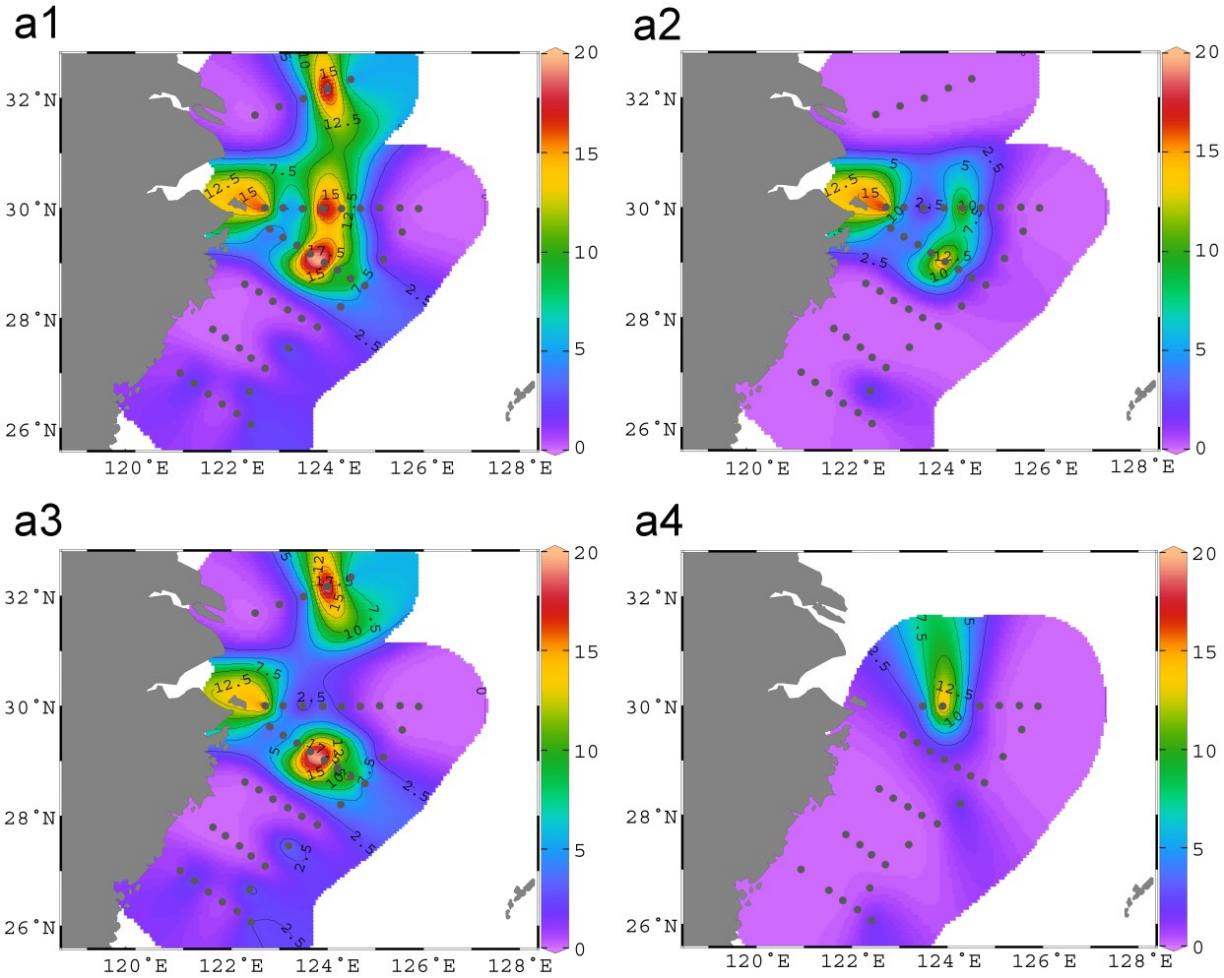
Horizontal distributions of *L*. *polyactis* in the East China Sea. Black dots indicate sampling stations. The x-axis and y-axis represent longitude and latitude, respectively. Concentrations of eDNA are depicted with gradient colors. Color bar scales indicate the log-transformed eDNA concentrations. (a1): average eDNA concentrations, (a2): eDNA concentrations in Layer1 (surface), (a3): eDNA concentrations in Layer2 (10m-50m), and (a4): eDNA concentrations in Layer3 (50m-104m). The eDNA hotspots were found in the Yushan fishing ground (sampling sites: S02-4, S02-5, S02-7), the Zhoushan fishing ground (S01-4, S01-1, S01-5), and the Dasha fishing ground (S00-4). New offshore spawning grounds (S01-5 and S02-5) were found.

The horizontal distributions of *L*. *polyactis* are presented in Fig 2 and S1 Table. The eDNA of *L*. *polyactis* was detected in 28 samples (16.37%) from 14 stations (31.82%). Statistical differences of eDNA concentration among stations were assessed using Kruskal-Wallis test. Significant differences (chi-squared = 79.923, df = 43, p = 0.0005332 < 0.01) were found in eDNA concentrations of *L*. *polyactis* among stations, indicating small yellow croaker exhibit apparent horizontal (regional) distributions. The eDNA hotspots were found in the Yushan fishing ground (sampling sites: S02-4, S02-5, S02-7), the Zhoushan fishing ground (S01-4, S01-1, S01-5), and the Dasha fishing ground (S00-4).

The biomass and distributions reflected by the eDNA were consistent with previously *L*. *polyactis* surveys with traditional methods on the spawning and feeding grounds [30–32]. The small yellow croaker is a migratory fish species that was considered one of the four fish species in traditional marine fisheries in China [4]. The overwintering *L*. *polyactis* adults begin their annual spawning migration toward the coastal waters in March. After spawning, the broodstocks migrate to the open area of the East China Sea for feeding, whereas larvae and juvenile fish continue to stay in the coastal nursery areas [31]. The spawning grounds of small yellow croaker are mostly located in western nearshore waters of the forbidden fish line. The spawning grounds along the southern Yellow Sea and the northern East China Sea are mainly located in and near the Changjiang Estuary, including the Lvsi fishing ground, the Changjiang fishing ground, and the Zhoushan fishing ground from early April to mid-May [30]. The spawning grounds along the East China Sea’s central and southern parts include the Yushan fishing ground and the Wentai fishing ground from late March to mid-April [30]. In short, the spawning ground of small yellow croaker is located in the coastal waters in spring [30]. Previous recruitment surveys showed the occurrence of young *L*. *polyactis* larvae in early May and a sharp density increase in mid-May to June [4, 33]. These field observations were supported by our results obtained from the eDNA approach, which strongly suggest eDNA is a powerful tool that can be used for small yellow croaker resource assessment, especially in motor-trawl prohibition areas and fishing forbidden seasons.

Traditionally, the spawning grounds of small yellow croaker are located in coastal areas, surrounding islands, estuaries, and semi-enclosed bays [31]. The Zhoushan, Yushan, and Lvsi fishing grounds (not included in our sampling area) were traditional spawning grounds of small yellow croaker based on ichthyoplankton surveys from motor-trawl capture data during 1971 to 1982 (since motor-trawl was forbidden in coastal areas) [30, 31]. The Western Dasha fishing ground and waters between the forbidden fishing line and the coastal line were traditional feeding grounds of small yellow croaker [30, 31]. However, recent studies found the small yellow croaker’s spawning grounds have obviously expanded from nearshore in the past to offshore at present [3, 6, 34]. Small yellow croaker is a demersal fish species and spawns pelagic eggs, which takes 40 days to develop from egg to post-flexion larvae [3]. Due to the lack of swimming ability, the larvae fish continued feeding near the spawning grounds [31, 32], and the high eDNA concentrations detected in offshore surface waters indicate offshore spawning grounds of small yellow croaker. Waters around S01-1, S01-5, and S02-5 with increased DNA concentrations indicate spawning grounds in these areas, of which S01-5 and S02-5 were offshore spawning grounds. The change of spawning grounds found previously is confirmed by our eDNA study. New offshore spawning grounds were found and verified in our current study.

Although trawling forbidden areas have been set along coastal areas to protect fishery resources, the spawning grounds of yellow croaker have expanded from nearshore to offshore areas. A likely explanation for this expansion is factors such as overfishing, climate change, and environment degradation might have caused spatial and temporal distributional changes in fish and consequently altered their habits and migration patterns [11, 12]. The novel eDNA approach offers a powerful tool for fish species conservation and management in addition to stock assessment [1]. Essential protection measures should be taken to avoid possible fishery depletion in small yellow croaker. As spawning grounds is important recruitment for fishery resources, it is thus recommended to add offshores fishing grounds into the current policies for the protection of spawning stocks and recruits.

### Vertical distribution of *L*. *polyactis*

**Fig 3:**
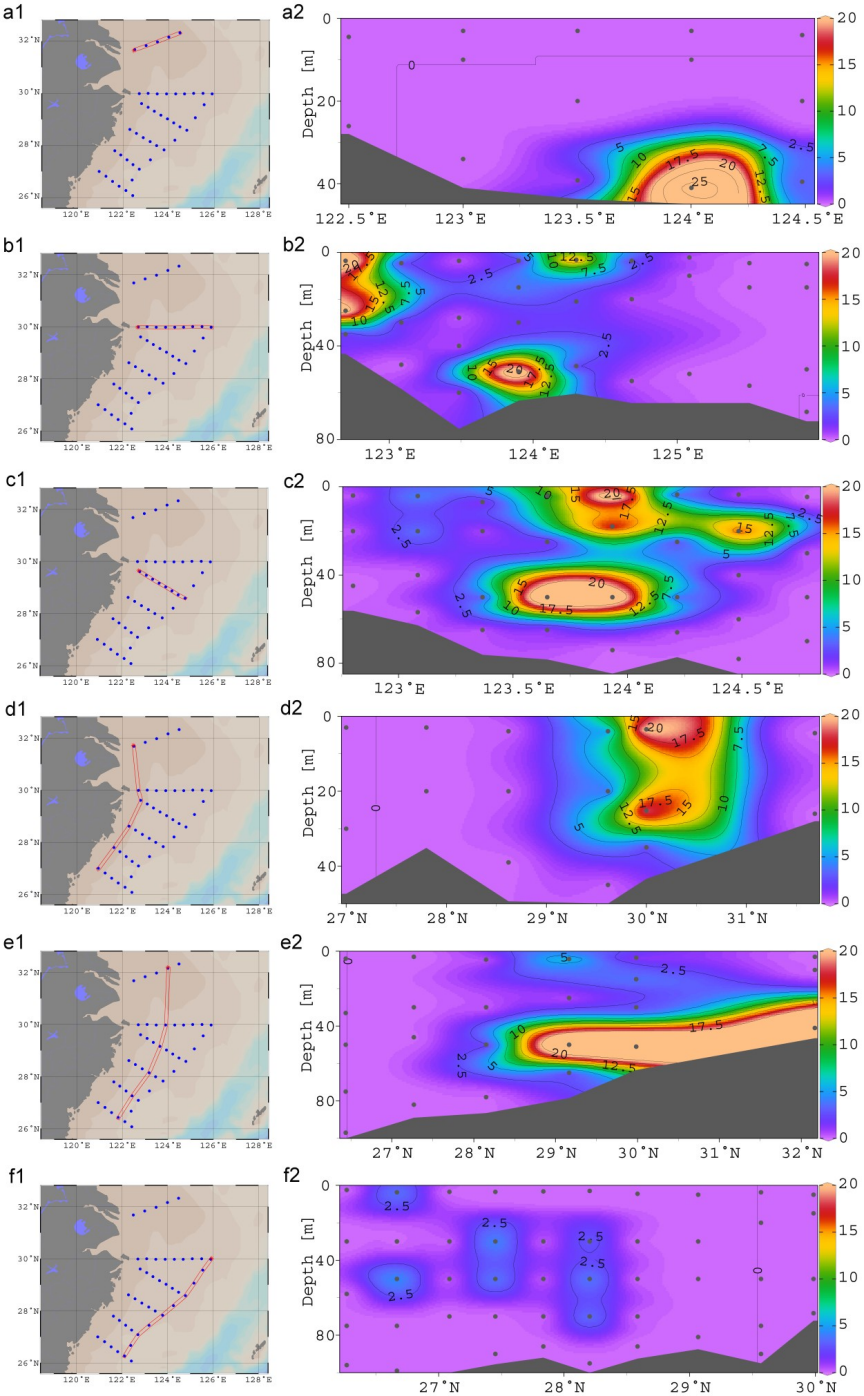
Vertical distributions of small yellow croaker along different longitude and latitude sections in the East China Sea. The x-axis and y-axis represent longitude and water depth, respectively, in a-c, whereas the x-axis and y-axis are latitude and water depth in d-f. Black dots denote sampling stations. The eDNA concentrations (logarithm transformed) are shown with gradient colors. A significant difference was found among three water layers of *L*. *polyactis* (chi-squared = 6.0571, df = 2, p = 0.04839 < 0.05).

**Fig 4:**
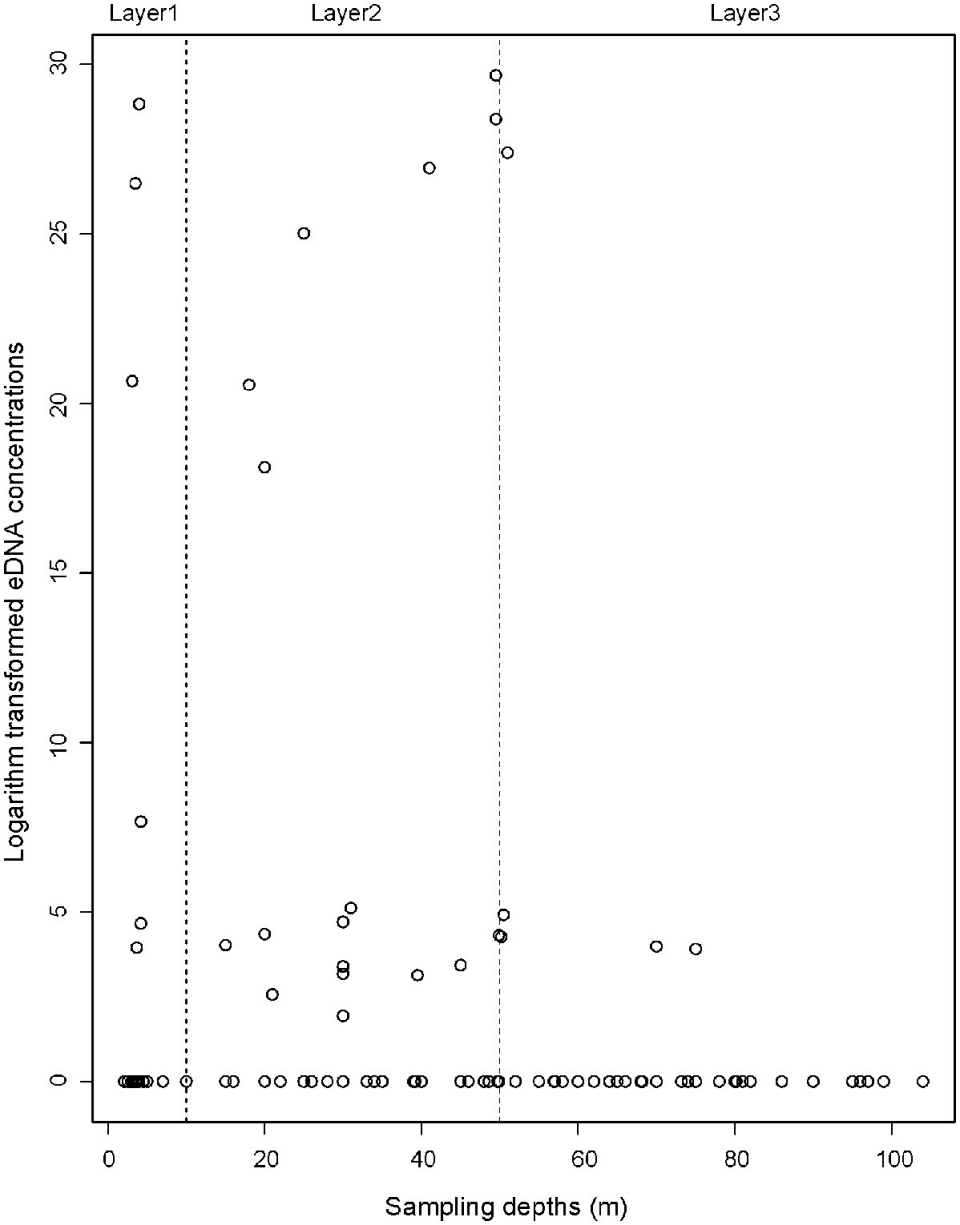
Vertical eDNA distributions of small yellow croaker in the East China Sea. The x-axis and y-axis represent sampling water depths and logarithm transformed eDNA concentrations, respectively. Circles denote sampling sites.

Layers were defined based on absolute sampling depths (i.e., Layer1: surface water; Layer2: 10-50m; and Layer3: 50m to the bottom). Vertical distributions of *L*. *polyactis* detected with eDNA were shown in Figs 3 and 4 and S1 Table. Statistical differences of eDNA concentration among layers were assessed using Kruskal-Wallis test. A significant difference was found among three water layers of *L*. *polyactis* (chi-squared = 6.0571, df = 2, p = 0.04839 < 0.05). Highest eDNA concentrations of *L*. *polyactis* were detected in S02-4 with a concentration of 7.81×10^12^ in layer 2, followed by S02-5 with 3.34×10^12^ in layer 1 and 2.16×10^12^ in layer 2. Concentrations larger than 1×10^7^ were found in S02-4, S02-5, S01-4, S00-4, S01-1, S01-5, S02-5, and S02-7. Average eDNA concentrations of layer 2 were 1.30×10^11^, followed by layer 1 (8.30×10^10^) and layer 3 (1.74×10^10^). The eDNA concentrations of *L*. *polyactis* in layer 2 was significantly higher than those in layer 3 (DF = 2, F = 0.4139, p = 0.046 < 0.05). No significant eDNA difference was found between surface water and middle water (p = 0.613 > 0.05). The eDNA residence time and ocean currents might influence the target organism detection. The eDNA concentration depends on its shedding from organism and decay in the environment. Previous studies reported eDNA can be detected and persist on short timescales and distance. For example, laboratory eDNA studies demonstrated that eDNA degraded below detection limits within few days in seawater [15, 35]. Field eDNA metabarcoding study also confirmed strong discrimination among diverse marine habitats connected by water movement [36]. These studies indicate the detectable DNA is mostly of local fish origin in the marine environment.

Due to the lack of movement ability, the hatching eggs and larvae were ichthyoplankton [3], so the eDNA detected in surface water were most likely from ichthyoplankton. Environmental DNA abundance could be contributed by larvae and juvenile *L*. *polyactis* in surface water and adult fish in the middle water. The eDNA was not detected in waters deeper than 30m around Zhoushan Archipelago, which strongly suggests only pelagic larval fish live in this area. The eDNA detected in layer 2 in the Dasha fishing ground is most likely from broodstocks aggregated for feeding. There were 3 samples that detected significant eDNA concentrations right at 50 m suggesting that small yellow croaker do inhabit waters at least as deep as 50 m and likely extend past this in certain areas. Of note, the concentrated aggregation detected in depth around 40m in certain sampling locations was not detected in the surface waters. This is in line with recent studies which showed eDNA dispersal was restricted by halocline [37] and pycnocline [38] using eDNA surveys. The vertical distribution of eDNA depends on the layer where the organism resides and the dispersion and degradation of eDNA [2]. So, this study suggests the broodstock biomass cannot be fully estimated using surface water eDNA abundance. Studies collecting water samples without considering the spawning or habitat preference will not provide precise distribution and abundance conclusion.

### Environmental variables related to the distribution of *L*. *polyactis*

The correlation was found to be significant between eDNA concentrations of *L*. *polyactis* and SiO_3_-Si.

**Fig 5:**
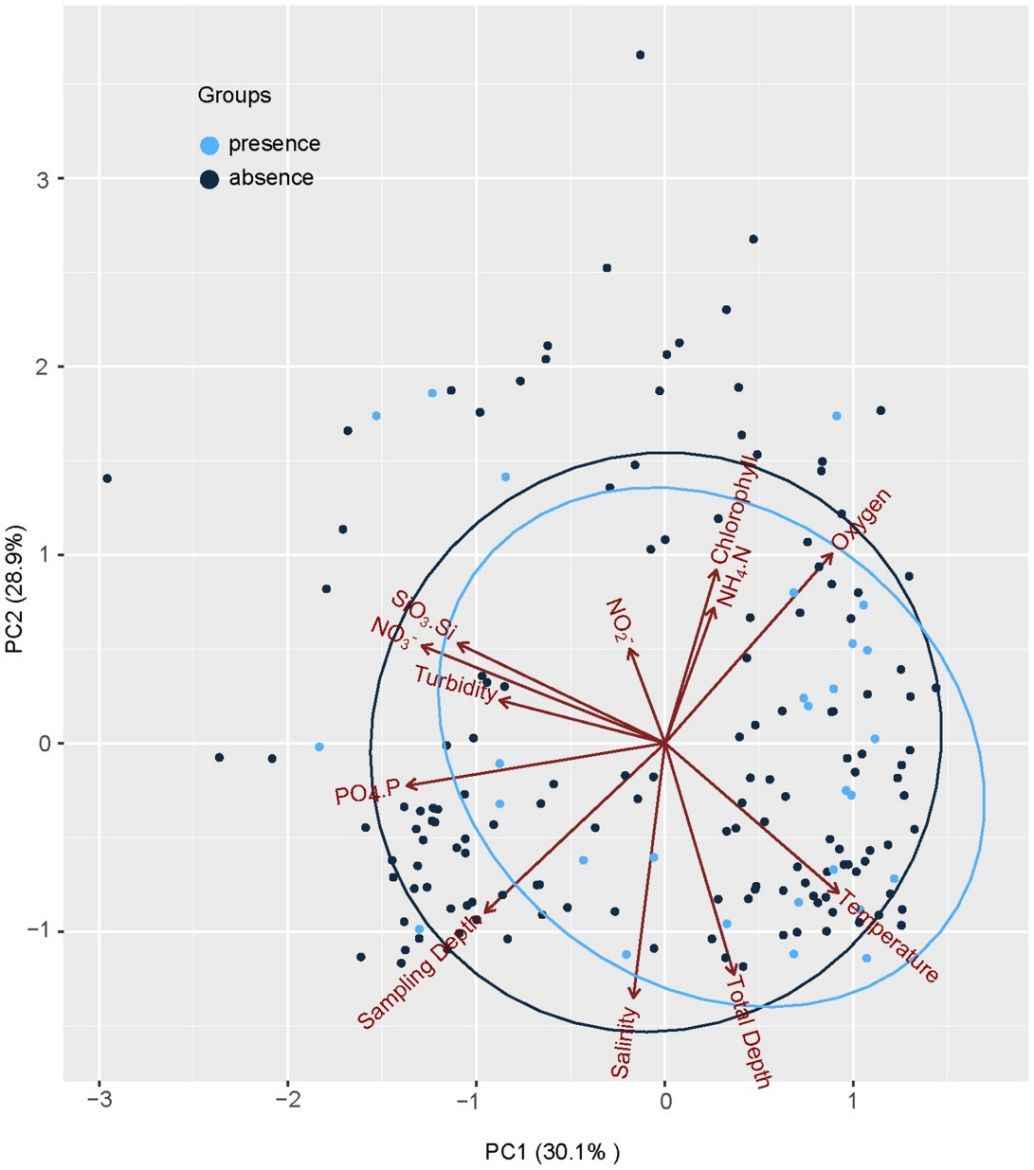
The Principal Component Analysis (PCA) results of environmental variables (total depth, sampling depth, temperature, salinity, chlorophyll, oxygen, turbidity, PO_4_.P, NH_4_.N, SiO_3_.Si, NO_3_^-^, and NO_2_^-^) of small yellow croaker sampling stations in the East China Sea. Dots indicate sampling stations, and circles indicate groups. Light blue: the presence of small yellow croaker detected by eDNA and dark blue: the absence of small yellow cracker, i.e., no species-specific eDNA detected. Two principal components were extracted and presented. PC1 explained 30.1%, and PC2 explained 29.8%. PC1 mostly represented nutrition, and PC2 represented sampling locations and layers in the current study.

In Fig 5, 12 variables were used for PCA analysis. The significance of each variable corresponds to the magnitude of the vector. The original 12 environmental variables of each sample were reduced into two principal components for which eigenvalues were > 1 and represented as a two-dimensional plot. PC1 explained 30.1%, and PC2 explained 28.9%. PC1 was mainly explained by NO3^-^ (0.91), SiO_3_-Si (0.80), PO_4_-P (0.78) and temperature (-0.75). PC2 was mainly explained by salinity (0.86), oxygen (-0.83), sampling depth (0.78), total depth (0.63), and chlorophyll (-0.63). So PC1 represented mainly nutrition and PC2 mostly represented locations and layers. As shown in Fig 5, the groupings of croaker presence and absence detected with eDNA were largely overlapped, indicating factors other than environmental variables may affect the distribution and aggregation behavior of small yellow croaker.

The MLR is a statistical technique that uses several explanatory variables to predict the outcome of a response variable. In the current study, the MLR was performed using all subsets regression method to evaluate whether any of the environmental variables could predict the abundance of small yellow croaker in the samples. We found that the MLR model for the eDNA concentrations of small yellow croaker could be best predicted by sampling depth (coefficient: 0.95, p = 0.03253 < 0.05), temperature (0.72, p = 0.1520 > 0.05), chlorophyll (0.58, p = 0.1981 > 0.05), NH_4_^-^.N (0.09, p = 0.02411 < 0.05), SiO_3_^-^.Si (1.45, p = 0.0001130 < 0.01), NO_3_^-^ (0.61, p = 0.007052 < 0.01) and an intercept (1.41e+05, p = 0.002890 < 0.01). The model could be useful and facilitate future surveys using eDNA or traditional methods.

## Conclusions

This study shows eDNA is a sensitive approach for the survey of distribution and the assessment of biomass of small yellow croaker in the East China Sea. Small yellow croaker was found to exhibit significant differences in horizontal distributions and varied vertical distributions with eDNA analysis, which suggests demonstrates the strategy of sampling both horizontally and vertically is essential for accurate detection and assessment of fishery resources. In addition, the results confirm previous findings regarding the distribution of *L*. *polyactis* and the expansion of nearshore spawning grounds. This study demonstrates the robustness of eDNA approach in monitoring *L*. *polyactis* at large horizontal and vertical scales. The developed protocols and major findings are expected to benefit long-term monitoring and protection programs and assist in sustainable fishery in small yellow croaker.

## Supporting information

S1 Table Sampling locations and eDNA concentrations of small yellow croaker. (DOCX)
