# Supplementary material for: Assessment of fishery resources using environmental DNA: Small yellow croaker (Larimichthys polyactis) in East China Sea
Source: PLoS One. 2020 Dec 29;15(12):e0244495. doi: 10.1371/journal.pone.0244495 (PMC7771671; doi:10.1371/journal.pone.0244495)
Supplement: S1 Table — (DOCX) [file pone.0244495.s001.docx]

S1 Table. Sampling locations and eDNA concentrations of small yellow croaker.

| station | longitude | latitude | Layer | YNLp | LpeDNA | lnLpeDNA |
| --- | --- | --- | --- | --- | --- | --- |
| S00-1 | 122.5 | 31.68333 | 1 | 0 | 0 | 0 |
| S00-1 | 122.5 | 31.68333 | 2 | 0 | 0 | 0 |
| S00-2 | 123.0003 | 31.83444 | 1 | 0 | 0 | 0 |
| S00-2 | 123.0003 | 31.83444 | 2 | 0 | 0 | 0 |
| S00-2 | 123.0003 | 31.83444 | 2 | 0 | 0 | 0 |
| S00-3 | 123.5011 | 31.98583 | 1 | 0 | 0 | 0 |
| S00-3 | 123.5011 | 31.98583 | 2 | 0 | 0 | 0 |
| S00-3 | 123.5011 | 31.98583 | 2 | 0 | 0 | 0 |
| S00-4 | 124.0003 | 32.16722 | 1 | 0 | 0 | 0 |
| S00-4 | 124.0003 | 32.16722 | 2 | 0 | 0 | 0 |
| S00-4 | 124.0003 | 32.16722 | 2 | 1 | 5.06E+11 | 26.9498 |
| S00-5 | 124.4853 | 32.33583 | 1 | 0 | 0 | 0 |
| S00-5 | 124.4853 | 32.33583 | 2 | 0 | 0 | 0 |
| S00-5 | 124.4853 | 32.33583 | 2 | 1 | 22 | 3.135494 |
| S01-1 | 122.6979 | 29.99815 | 1 | 1 | 3.23E+11 | 26.50092 |
| S01-1 | 122.6979 | 29.99815 | 2 | 1 | 7.38E+10 | 25.02397 |
| S01-1 | 122.6979 | 29.99815 | 2 | 0 | 0 | 0 |
| S01-2 | 123.0858 | 30.00028 | 1 | 0 | 0 | 0 |
| S01-2 | 123.0858 | 30.00028 | 2 | 0 | 0 | 0 |
| S01-2 | 123.0858 | 30.00028 | 2 | 0 | 0 | 0 |
| S01-3 | 123.4858 | 29.98583 | 1 | 0 | 0 | 0 |
| S01-3 | 123.4858 | 29.98583 | 2 | 0 | 0 | 0 |
| S01-3 | 123.4858 | 29.98583 | 2 | 0 | 0 | 0 |
| S01-3 | 123.4858 | 29.98583 | 3 | 0 | 0 | 0 |
| S01-4 | 123.9003 | 29.98528 | 1 | 0 | 0 | 0 |
| S01-4 | 123.9003 | 29.98528 | 2 | 1 | 55 | 4.025352 |
| S01-4 | 123.9003 | 29.98528 | 2 | 1 | 5.94604 | 1.938172 |
| S01-4 | 123.9003 | 29.98528 | 3 | 1 | 7.99E+11 | 27.40663 |
| S01-5 | 124.3 | 29.98583 | 1 | 1 | 9.47E+08 | 20.66861 |
| S01-5 | 124.3 | 29.98583 | 2 | 0 | 12 | 2.564949 |
| S01-5 | 124.3 | 29.98583 | 2 | 0 | 0 | 0 |
| S01-6 | 124.6833 | 29.98583 | 1 | 0 | 0 | 0 |
| S01-6 | 124.6833 | 29.98583 | 2 | 0 | 0 | 0 |
| S01-6 | 124.6833 | 29.98583 | 3 | 0 | 0 | 0 |
| S01-7 | 125.0856 | 30 | 1 | 0 | 0 | 0 |
| S01-7 | 125.0856 | 30 | 2 | 0 | 0 | 0 |
| S01-7 | 125.0856 | 30 | 3 | 0 | 0 | 0 |
| S01-8 | 125.5 | 30.00028 | 1 | 0 | 0 | 0 |
| S01-8 | 125.5 | 30.00028 | 2 | 0 | 0 | 0 |
| S01-8 | 125.5 | 30.00028 | 3 | 0 | 0 | 0 |
| S01-9 | 125.9 | 29.98556 | 1 | 0 | 0 | 0 |
| S01-9 | 125.9 | 29.98556 | 2 | 0 | 0 | 0 |
| S01-9 | 125.9 | 29.98556 | 2 | 0 | 0 | 0 |
| S01-9 | 125.9 | 29.98556 | 3 | 0 | 0 | 0 |
| S02-1 | 122.8014 | 29.61694 | 1 | 0 | 0 | 0 |
| S02-1 | 122.8014 | 29.61694 | 2 | 0 | 0 | 0 |
| S02-1 | 122.8014 | 29.61694 | 2 | 0 | 0 | 0 |
| S02-2 | 123.0842 | 29.46722 | 1 | 1 | 105 | 4.663439 |
| S02-2 | 123.0842 | 29.46722 | 2 | 1 | 77 | 4.356709 |
| S02-2 | 123.0842 | 29.46722 | 2 | 0 | 0 | 0 |
| S02-2 | 123.0842 | 29.46722 | 3 | 0 | 0 | 0 |
| S02-3 | 123.3675 | 29.31778 | 1 | 0 | 0 | 0 |
| S02-3 | 123.3675 | 29.31778 | 2 | 0 | 0 | 0 |
| S02-3 | 123.3675 | 29.31778 | 2 | 0 | 0 | 0 |
| S02-3 | 123.3675 | 29.31778 | 3 | 0 | 0 | 0 |
| S02-4 | 123.65 | 29.16722 | 1 | 1 | 2138 | 7.668094 |
| S02-4 | 123.65 | 29.16722 | 2 | 0 | 0 | 0 |
| S02-4 | 123.65 | 29.16722 | 2 | 1 | 7.81E+12 | 29.68643 |
| S02-4 | 123.65 | 29.16722 | 3 | 0 | 0 | 0 |
| S02-5 | 123.9342 | 29.01722 | 1 | 1 | 3.34E+12 | 28.83699 |
| S02-5 | 123.9342 | 29.01722 | 2 | 1 | 8.46E+08 | 20.55565 |
| S02-5 | 123.9342 | 29.01722 | 2 | 1 | 2.16E+12 | 28.40113 |
| S02-5 | 123.9342 | 29.01722 | 3 | 0 | 0 | 0 |
| S02-6 | 124.2172 | 28.87084 | 1 | 0 | 0 | 0 |
| S02-6 | 124.2172 | 28.87084 | 2 | 0 | 0 | 0 |
| S02-6 | 124.2172 | 28.87084 | 2 | 0 | 0 | 0 |
| S02-6 | 124.2172 | 28.87084 | 3 | 0 | 0 | 0 |
| S02-7 | 124.4858 | 28.7175 | 1 | 0 | 0 | 0 |
| S02-7 | 124.4858 | 28.7175 | 2 | 1 | 74139764 | 18.12146 |
| S02-7 | 124.4858 | 28.7175 | 2 | 0 | 0 | 0 |
| S02-7 | 124.4858 | 28.7175 | 3 | 0 | 0 | 0 |
| S02-7 | 124.4858 | 28.7175 | 3 | 0 | 0 | 0 |
| S02-8 | 124.7853 | 28.58361 | 1 | 0 | 0 | 0 |
| S02-8 | 124.7853 | 28.58361 | 2 | 0 | 0 | 0 |
| S02-8 | 124.7853 | 28.58361 | 2 | 0 | 0 | 0 |
| S02-8 | 124.7853 | 28.58361 | 3 | 0 | 0 | 0 |
| S02-8 | 124.7853 | 28.58361 | 3 | 0 | 0 | 0 |
| S03-1 | 122.2833 | 28.61861 | 1 | 0 | 0 | 0 |
| S03-1 | 122.2833 | 28.61861 | 2 | 0 | 0 | 0 |
| S03-1 | 122.2833 | 28.61861 | 2 | 0 | 0 | 0 |
| S03-2 | 122.5692 | 28.46722 | 1 | 0 | 0 | 0 |
| S03-2 | 122.5692 | 28.46722 | 2 | 0 | 0 | 0 |
| S03-2 | 122.5692 | 28.46722 | 2 | 0 | 0 | 0 |
| S03-2 | 122.5692 | 28.46722 | 3 | 0 | 0 | 0 |
| S03-3 | 122.8692 | 28.30111 | 1 | 0 | 0 | 0 |
| S03-3 | 122.8692 | 28.30111 | 2 | 0 | 0 | 0 |
| S03-3 | 122.8692 | 28.30111 | 3 | 0 | 0 | 0 |
| S03-3 | 122.8692 | 28.30111 | 3 | 0 | 0 | 0 |
| S03-4 | 123.1692 | 28.15167 | 1 | 0 | 0 | 0 |
| S03-4 | 123.1692 | 28.15167 | 2 | 0 | 0 | 0 |
| S03-4 | 123.1692 | 28.15167 | 2 | 0 | 0 | 0 |
| S03-4 | 123.1692 | 28.15167 | 3 | 0 | 0 | 0 |
| S03-5 | 123.4686 | 28 | 1 | 0 | 0 | 0 |
| S03-5 | 123.4686 | 28 | 2 | 0 | 0 | 0 |
| S03-5 | 123.4686 | 28 | 2 | 0 | 0 | 0 |
| S03-5 | 123.4686 | 28 | 3 | 0 | 0 | 0 |
| S03-5 | 123.4686 | 28 | 3 | 0 | 0 | 0 |
| S03-6 | 123.7833 | 27.83444 | 1 | 0 | 0 | 0 |
| S03-6 | 123.7833 | 27.83444 | 2 | 0 | 0 | 0 |
| S03-6 | 123.7833 | 27.83444 | 2 | 0 | 0 | 0 |
| S03-6 | 123.7833 | 27.83444 | 3 | 0 | 0 | 0 |
| S03-6 | 123.7833 | 27.83444 | 3 | 0 | 0 | 0 |
| S04-1 | 121.6181 | 27.80028 | 1 | 0 | 0 | 0 |
| S04-1 | 121.6181 | 27.80028 | 2 | 0 | 0 | 0 |
| S04-2 | 121.8856 | 27.63333 | 1 | 0 | 0 | 0 |
| S04-2 | 121.8856 | 27.63333 | 2 | 0 | 0 | 0 |
| S04-2 | 121.8856 | 27.63333 | 2 | 0 | 0 | 0 |
| S04-2 | 121.8856 | 27.63333 | 3 | 0 | 0 | 0 |
| S04-3 | 122.1672 | 27.45 | 1 | 0 | 0 | 0 |
| S04-3 | 122.1672 | 27.45 | 2 | 0 | 0 | 0 |
| S04-3 | 122.1672 | 27.45 | 2 | 0 | 0 | 0 |
| S04-3 | 122.1672 | 27.45 | 3 | 0 | 0 | 0 |
| S04-4 | 122.4186 | 27.26889 | 1 | 0 | 0 | 0 |
| S04-4 | 122.4186 | 27.26889 | 2 | 0 | 0 | 0 |
| S04-4 | 122.4186 | 27.26889 | 2 | 0 | 0 | 0 |
| S04-4 | 122.4186 | 27.26889 | 3 | 0 | 0 | 0 |
| S04-5 | 122.7006 | 27.08556 | 1 | 0 | 0 | 0 |
| S04-5 | 122.7006 | 27.08556 | 2 | 0 | 0 | 0 |
| S04-5 | 122.7006 | 27.08556 | 2 | 0 | 0 | 0 |
| S04-5 | 122.7006 | 27.08556 | 3 | 0 | 0 | 0 |
| S04-5 | 122.7006 | 27.08556 | 3 | 0 | 0 | 0 |
| S05-1 | 120.9336 | 27 | 1 | 0 | 0 | 0 |
| S05-1 | 120.9336 | 27 | 2 | 0 | 0 | 0 |
| S05-1 | 120.9336 | 27 | 2 | 0 | 0 | 0 |
| S05-1 | 120.9336 | 27 | 3 | 0 | 0 | 0 |
| S05-2 | 121.2333 | 26.81722 | 1 | 0 | 0 | 0 |
| S05-2 | 121.2333 | 26.81722 | 2 | 0 | 0 | 0 |
| S05-2 | 121.2333 | 26.81722 | 2 | 1 | 166 | 5.117994 |
| S05-3 | 121.5186 | 26.61861 | 1 | 0 | 0 | 0 |
| S05-3 | 121.5186 | 26.61861 | 2 | 0 | 0 | 0 |
| S05-3 | 121.5186 | 26.61861 | 2 | 0 | 0 | 0 |
| S05-3 | 121.5186 | 26.61861 | 3 | 0 | 0 | 0 |
| S05-4 | 121.8172 | 26.43583 | 1 | 0 | 0 | 0 |
| S05-4 | 121.8172 | 26.43583 | 2 | 0 | 0 | 0 |
| S05-4 | 121.8172 | 26.43583 | 2 | 0 | 0 | 0 |
| S05-4 | 121.8172 | 26.43583 | 3 | 0 | 0 | 0 |
| S05-4 | 121.8172 | 26.43583 | 3 | 0 | 0 | 0 |
| S05-5 | 122.1175 | 26.26833 | 1 | 0 | 0 | 0 |
| S05-5 | 122.1175 | 26.26833 | 2 | 0 | 0 | 0 |
| S05-5 | 122.1175 | 26.26833 | 3 | 0 | 0 | 0 |
| S05-5 | 122.1175 | 26.26833 | 3 | 0 | 0 | 0 |
| S05-5 | 122.1175 | 26.26833 | 3 | 0 | 0 | 0 |
| S05-6 | 122.4172 | 26.06806 | 1 | 0 | 0 | 0 |
| S05-6 | 122.4172 | 26.06806 | 2 | 1 | 29 | 3.401197 |
| S05-6 | 122.4172 | 26.06806 | 2 | 1 | 30 | 3.433987 |
| S05-6 | 122.4172 | 26.06806 | 3 | 1 | 49 | 3.912023 |
| S05-6 | 122.4172 | 26.06806 | 3 | 0 | 0 | 0 |
| S12-1 | 125.55 | 29.56917 | 1 | 0 | 0 | 0 |
| S12-1 | 125.55 | 29.56917 | 2 | 0 | 0 | 0 |
| S12-1 | 125.55 | 29.56917 | 2 | 0 | 0 | 0 |
| S12-1 | 125.55 | 29.56917 | 3 | 0 | 0 | 0 |
| S12-1 | 125.55 | 29.56917 | 3 | 0 | 0 | 0 |
| S12-2 | 125.1689 | 29.06722 | 1 | 0 | 0 | 0 |
| S12-2 | 125.1689 | 29.06722 | 2 | 0 | 0 | 0 |
| S12-2 | 125.1689 | 29.06722 | 2 | 0 | 0 | 0 |
| S12-2 | 125.1689 | 29.06722 | 3 | 0 | 0 | 0 |
| S23-1 | 124.2686 | 28.2025 | 1 | 0 | 0 | 0 |
| S23-1 | 124.2686 | 28.2025 | 2 | 1 | 23 | 3.178054 |
| S23-1 | 124.2686 | 28.2025 | 2 | 1 | 74 | 4.317488 |
| S23-1 | 124.2686 | 28.2025 | 3 | 1 | 53 | 3.988984 |
| S23-1 | 124.2686 | 28.2025 | 3 | 0 | 0 | 0 |
| S34-1 | 123.1847 | 27.45167 | 1 | 0 | 0 | 0 |
| S34-1 | 123.1847 | 27.45167 | 2 | 1 | 110.0877 | 4.71032 |
| S34-1 | 123.1847 | 27.45167 | 2 | 1 | 71 | 4.276666 |
| S34-1 | 123.1847 | 27.45167 | 3 | 0 | 0 | 0 |
| S34-1 | 123.1847 | 27.45167 | 3 | 0 | 0 | 0 |
| S45-1 | 122.3842 | 26.66861 | 1 | 1 | 51 | 3.951244 |
| S45-1 | 122.3842 | 26.66861 | 2 | 0 | 0 | 0 |
| S45-1 | 122.3842 | 26.66861 | 2 | 1 | 136 | 4.919981 |
| S45-1 | 122.3842 | 26.66861 | 3 | 0 | 0 | 0 |
| S45-1 | 122.3842 | 26.66861 | 3 | 0 | 0 | 0 |

Layers were defined based on absolute sampling depths (i.e., Layer1: surface water; Layer2: 10-50m; and Layer3: 50m to the bottom); YNLp indicated eDNA presence/absence of small yellow croaker; LpeDNA indicated eDNA concentrations (copies/L) of small yellow croaker; and lnLpeDNA indicated logarithm transformed eDNA concentrations (i.e., ln([eDNA]+1)).
